# Supplementary material for: New insight into the molecular mechanism of colour differentiation among floral segments in orchids
Source: Commun Biol. 2020 Feb 28;3:89. doi: 10.1038/s42003-020-0821-8 (PMC7048853; doi:10.1038/s42003-020-0821-8)
Supplement: Supplementary file 1 — Supplementary Information [file 42003_2020_821_MOESM1_ESM.pdf]

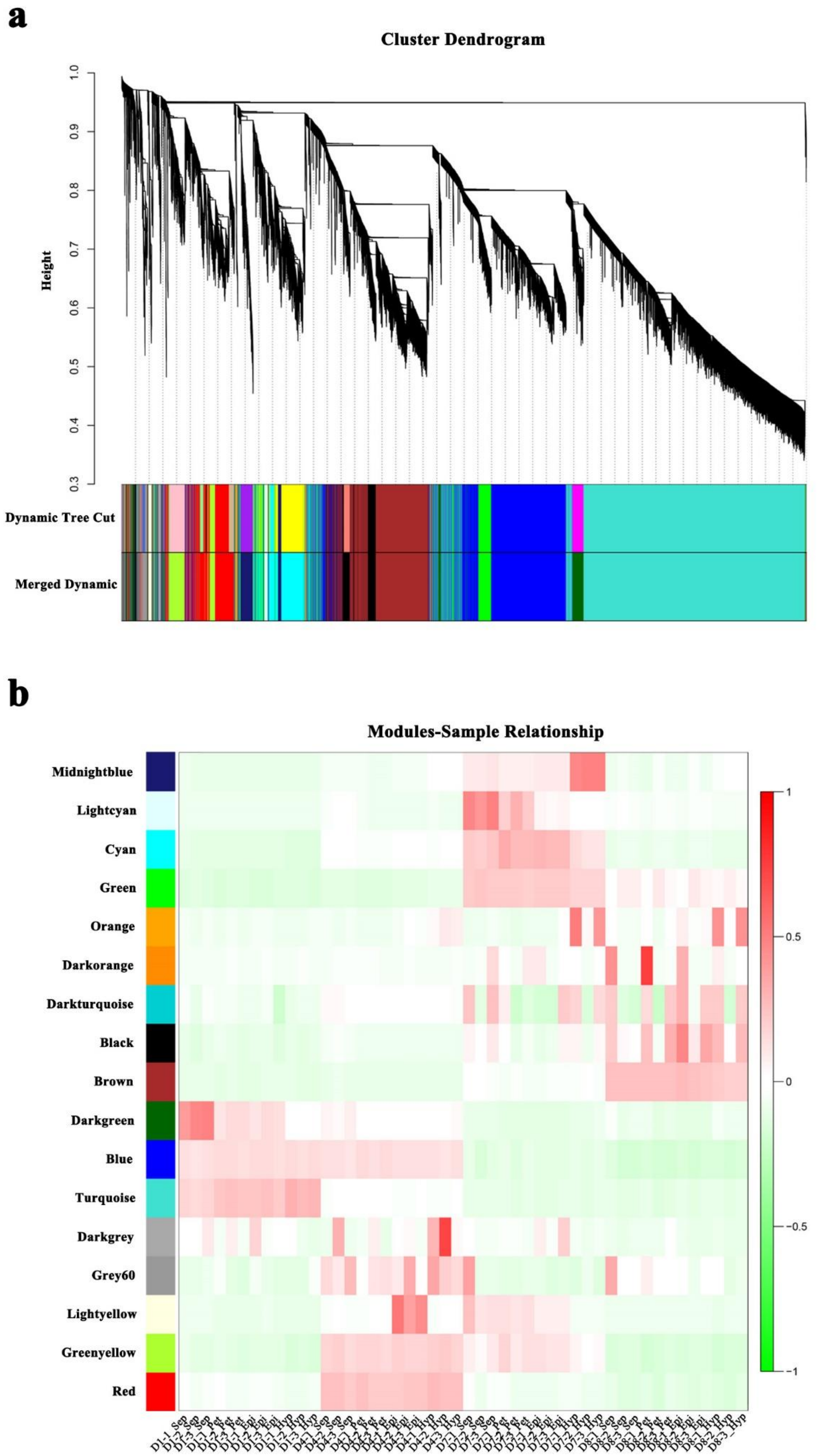

Supplemental Figure 1. Weighted gene co-expression network analysis (WGCNA)

in the sepals/petals and lip segments during KOVA flower development. a, Hierarchical clustering tree of isoforms/unigenes based on their expression level in 48 transcriptomes. Each branch represents an isoform or unigene, and each colour below represents a module. The dynamic tree cut shows that the isoforms/unigenes fell into different modules. The merged dynamic indicates that the modules were divided by clustering modules with similar expression patterns. **b**, The heatmap analysis displays the module expression pattern in the 48 samples. Sepal, petal, epichile, and hypochile are abbreviated Sep, Pet, Epi, and Hyp, respectively. Each Sep, Pet, Epi, and Hyp in the four stages were sampled with three biological replicates.

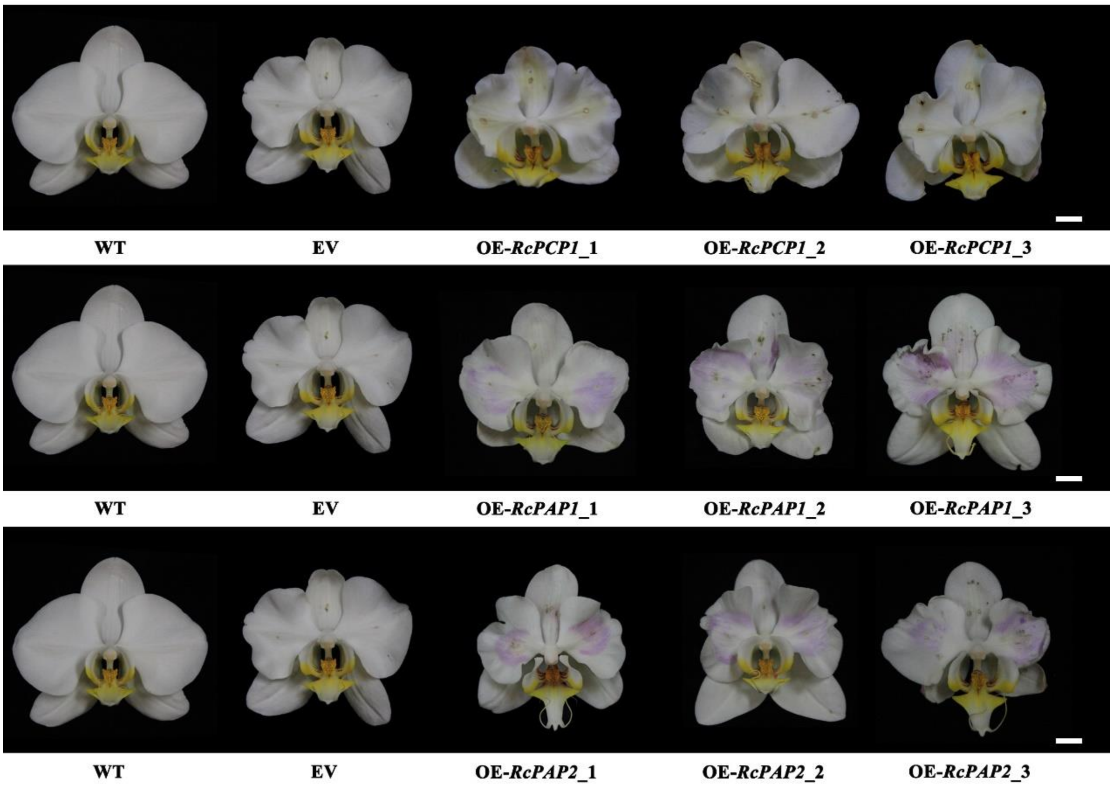

Supplemental Figure 2. The transient overexpression essays of *RcPCP1*, *RcPAP1* and *RcPAP2*. To ensure the reliability of the results, the phenotype of the transient overexpression assay of each isoform/unigene was independently repeated three times

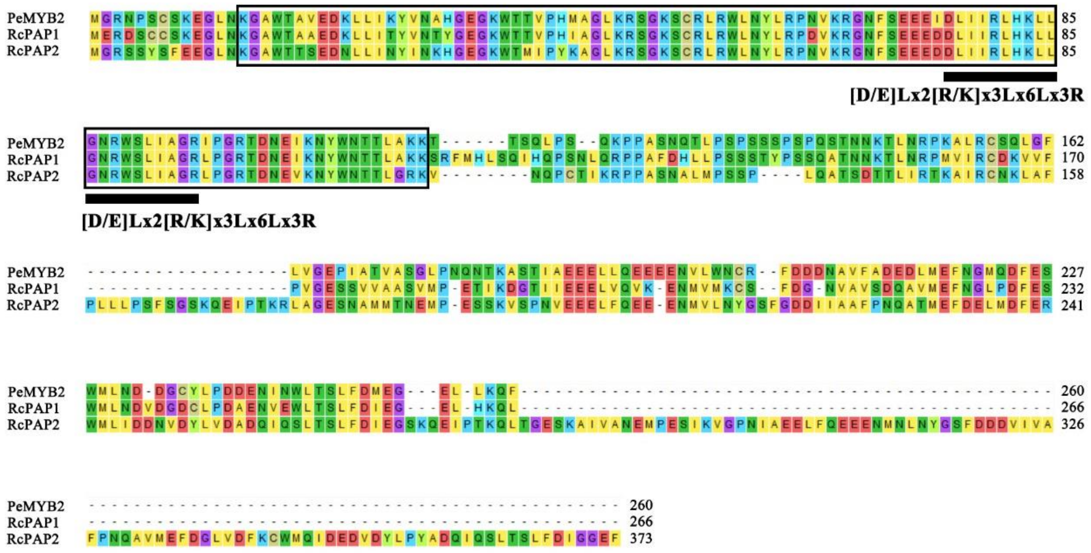

Supplemental Figure 3. Alignment of the amino acid sequence of RcPAP1, RcPAP2 and PeMYB2. These three MYBs have their own specific amino acid sequence, especially in positions from 200 to 372 bp. The DNA-binding domain of R2R3 MYB is labeled by the black box and the motif ([D/E]Lx2[R/K]x3Lx6Lx3R) that interacts with a bHLH transcription factor in *Arabidopsis*[1] is indicated by a black bar below the alignment.

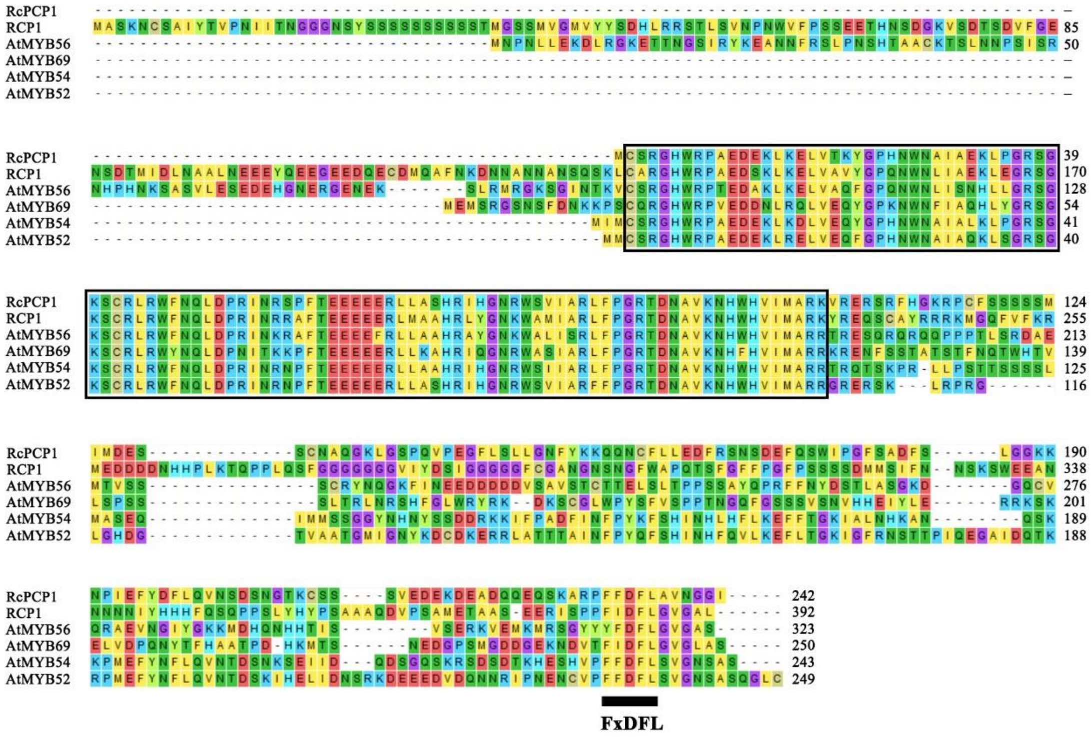

Supplemental Figure 4. Alignment of the amino acid sequence of RcPCP1, RCP1 and the closely related homologs in *Arabidopsis thaliana*. The DNA-binding domain of R2R3 MYB is labeled by the black box and the signature motif (FxDFL) that defines the Subgroup 21 R2R3 MYB clade[2] is indicated by a black bar below the alignment.

Supplementary Table 1. Primers used in this study.

For qRT-PCR	
Primer name	Sequence (5'-3')
18S_F	AAGCTCGCAAGTACACGACT
18S_R	TCCAATGGTGCTCGTTCTCA
RcPAP1_F	ACGTGGAAACTTCTCCGAGG
RcPAP1_R	AGATAGATGCATGAAGCGCGA
RcPAP2_F	ATGGCTGAACTACCTGAGGC
RcPAP2_R	TGGTGCATGGTTGATTCACCT
RcPCP1_F	ACGCAATCGCTGAGAAGCTC
RcPCP1_R	CTTCACAGCGTTATCTGTGCG
BCH1_F	TAAGAGTGGCGCAGTGATCG
BCH1_R	CTGGACATTATCGCCGCAAC
AP3-1_F	GAGCTCACTGTTCTCTGCGA
AP3-1_R	GAGATGCTTCAGTGTATGCTGC
AP3-2_F	CCTACCGCTGACACCAAGAG
AP3-2_R	CTCAAGACCACGCAGTTCCT
AP3-3_F	GCCGGCAAGTAACGTACTCA
AP3-3_R	ATGCCGGTGACTTGCTGATA
AP3-4_F	CTCTCAAGAAGCACACCGGA
AP3-4_R	AGATCATGCGAGCCGTATCC
AGL6-1_F	CCTTCTCCAAGCGCAGGAAT
AGL6-1_R	ACTCATAGAGCTTGCCTCGG
AGL6-2_F	TTGGAACAGACGGTGGATCG
AGL6-2_R	CCAACTCCACCATTCCTCGG
PeActin_4_F	TTGTGAGCAACTGGGATGACAT
PeActin_4_R	GCCACGCGAAGTTCATTGT
PePSY_F	CGGAGAGGAGACGAGCAATC
PePSY_R	GCTGGATGTCGACAGGGTAG

PePDS_F	AGTGGATGAAACGGCAGGG
PePDS_R	TGCATAGCCTCTCAGGAGGA
PeZ-ISO_F	GTGAGAAGCTCATTGGCGAG
PeZ-ISO_R	CCACACAAGCTCATGAACCC
PeZDS_F	GTCCCGTTGTTCGAGCTCTT
PeZDS_R	GCAAGAGAGAAGCCTCGGTT
PeCRTISO_F	CATGTCTGGTGCTGCAATGG
PeCRTISO_R	CCAGCGAGTCAAGGATCTCA
PeLCYE_F	TCGCTTGCCTGATTGGATGT
PeLCYE_R	AAGCCAGACGGATCAGCAAA
PeLCYB_F	CGCCTTCGTCTACATCGACA
PeLCYB_R	GTGGACGACGTTGAGGACTT
PeBCH1_F	GATTTGTTGGCGATGCGGAG
PeBCH1_R	TCCGTCAATGGAACTTCGCT
PeCHS_F	ACTGCGAGCATCTCACTGAC
PeCHS_R	GACCTCGGCGACAACTATGT
PeCHI_F	TCGGAGTGTACTTGGAAGCG
PeCHI_R	CCGTTAATGGCAGCAGCATC
PeF3H_F	TGGAGATGGAGGTCGAAGGT
PeF3H_R	CGGCAGCAGATCGAAGAACT
PeF3’H_F	CGGAACCTGGACTACCATCG
PeF3’H_R	AATTACGGAATGACGGCGGA
PeF3’5’H_F	TCATTACCTGAGCAACGGTCGG
PeF3’5’H_R	AACGCCGCGATCGAAAGCC
PeDFR_F	AGGATATGTTTGATGATGCTATTAAG
PeDFR_R	TTCCTCAGTGTTGGGCGGTAAGAG
PeANS_F	TGCCACAGCGTTCTTCACCG
PeANS_R	AAACCGCCCACGAGATCCGTAC
For Isoform/Unigene CDS clone	

RcPAP1_CDS_F	CCCTCGAGGGATGGAAAGGGATTCATGTTGT
RcPAP1_CDS _R	CCCTCGAGGGTTATAATTGCTTATGCAGCTC
RcPAP2_CDS _F	CCCTCGAGGGATGGGAAGGAGTTCTTACTCT
RcPAP2_CDS _R	CCCTCGAGGGCTAGAATTCACCTCCTATATC
RcPCP1_CDS _F	CCCTCGAGGGATGTGTTCAAGAGGTCATTGG
RcPCP1_CDS _R	CCCTCGAGGGTTAAATGCCCCCATTAACAGC

Supplementary reference:

1. Zimmermann, I. M. et al. Comprehensive identification of *Arabidopsis thaliana* MYB transcription factors interacting with R/B-like BHLH proteins. *Plant J*. **40**, 22–34 (2004).
2. Stracke, R. et al. The *R2R3-MYB* gene family in *Arabidopsis thaliana*. *Curr. Opin. Plant Biol*. **4**, 447–456 (2001).
